# Reinforced Olive Pâté as a Source of Antioxidants with Positive Effects on Young Smokers

**DOI:** 10.3390/medicina55100680

**Published:** 2019-10-09

**Authors:** Pierpaolo Cavallo, Francesco Vinale, Immacolata Sepe, Gennaro Galasso, Francesca Luisa Fedele, Andrea Sicari, Loredana Zito, Matteo Lorito

**Affiliations:** 1 Dipartimento di Fisica “E.R.Caianiello”/DF, Università di Salerno, Via Giovanni Paolo II, 132, 84084 Fisciano (SA), Italy; ggalasso@unisa.it; 2 ISC-CNR, Piazzale Aldo Moro, 5, 00185 Rome, Italy; 3 Dipartimento di Agraria, Università di Napoli “Federico II”, Via Università, 96, 80055 Portici (NA), Italy; frvinale@unina.it (F.V.); lorito@unina.it (M.L.); 4 Diagnostica Cavallo—Centro Ricerca, Via C. Calo’, 2, 84123 Salerno, Italy; immasepe@gmail.com; 5 Linfa Scarl, Via Ferrante Imparato 27/29, 80100 Napoli, Italy; francalisa@yahoo.com (F.L.F.); andrea@laboratoriolinfa.it (A.S.); 6 Santa Rita Srl, Via Zona Industriale, 89900 Vibo Valentia, Italy; lo.zito987@gmail.com

**Keywords:** antioxidants, nutrition, functional foods, smokers, olive oil, olive derived foods

## Abstract

*Background and objectives*: Olive pâté (OP) is an olive-derived product with potentially beneficial effects on human health due to the presence of natural antioxidants. The present dietary supplementation study aimed to evaluate the effects on blood antioxidant levels of an olive pâté reinforced with natural antioxidants (ROP) recovered from olive mill waste. *Materials and methods*: Ninety-eight healthy volunteers (M = 54, 55%, age 18–25) were divided into two groups: A (*n* = 49), practicing three or more days of physical activity a week, and B (*n* = 49), practicing less than two. Each group was split into two subgroups, receiving dietary supplementation with OP or ROP. The status of smoker was also recorded, and a biological antioxidant potential (BAP) test was performed on each subject. *Results*: The BAP values increased with both OP (*n* = 30) and ROP (*n* = 68) but ROP supplementation showed higher increments (736.9 μmol/L) than OP (339.6). The increment was significantly higher for smokers (*n* = 15), 1122.9 vs. non-smokers (*n* = 53), 635.7, with values in percent of baseline, respectively, 34.6% and 16.2% (*P* < 0.01). *Conclusions*: The ROP nutritional supplementation appears useful to increase antioxidant activity, with better effect in smokers; further studies should confirm the finding and investigate its biological bases.

## 1. Introduction

Extra virgin olive oil (EVOO) is a highly valued product and is one of the main components of the “Mediterranean Diet”, which includes a group of about 15 countries with peculiar foods, habits, and cultures. [1]

EVOO shows a health-promoting action due to its profile of phenolic fractions, including phenols (hydroxytyrosol and tyrosol), secoiridoids, and lignans. [2] Its production process includes crushing in a hammer mill to obtain the “pomace”, that is a mixture of the crushed olive pericarp and stone, then pressed to extract a liquid mix of oil and water, the olive mill waste water (OMWW), which are finally separated to obtain the clear oil.

The by-product OMWW is a rich source of natural antioxidants, which can be recovered from it and used to “reinforce” the antioxidant levels in food, which may lead to an increase in olive oil phenols level [3] in the diet, as their absorption in humans is dose-dependent [4].

During the last few years, OMWW has attracted increasing attention as a source of high added-value compounds, such as sugars and minerals, useful as cosmeceutical ingredients [5], and biophenols, well known for their health effects due to antioxidant and anti-inflammatory activities [6]. These compounds can be recovered using green extraction methods [7] and used to “reinforce” the antioxidant levels in food, which may lead to an increase in olive oil phenols level in the diet, as their absorption in humans is dose-dependent.

There are also many olive-derived foods in the Mediterranean gastronomy tradition: one is olive pâté (OP) [8] which is prepared using only the pericarp (named “olive fruit paste”) used for gastronomy, and increasingly being recognized [9] for its beneficial effects on human health. Some studies on its chemical characteristics have been published, but no data appear to be available on its antioxidant activity.

Generally speaking, many factors connected to lifestyle and persona behavior influence the oxidative status in a healthy subject. Among these factors, cigarette smoke [10] induces systemic oxidative stress, which could be involved in the etiology of chronic and neoplastic diseases, while [11,12,13] regular and moderate exercise positively influences systemic oxidative status.

The rationale of the present study is that adding an olive pâté to the usual nutrition, an increase in the antioxidant levels is expected; moreover, if the pâté is reinforced with a source of natural antioxidants, possibly recovered from OMWW, the resulting reinforced olive pâté (ROP) should assure an increase in antioxidants levels higher than that obtained with the basic OP.

The present study aimed at evaluating the effects of dietary supplementation in healthy volunteers, comparing the antioxidant potential of healthy subjects aged 18 to 25, with different levels of physical activity, before and after 30 days of dietary supplementation with OP or ROP. This time duration was chosen due to the limited ROP quantity available.

## 2. Materials and Methods

### 2.1. OP and ROP Preparation

OP preparation included washing, separation of pericarp from stone, smashing, and mixing with olive oil, packaging, pasteurization, and quality control. ROP was prepared by adding the commercial OMWW extract of *Olea europea* (cv Carolea) fruit obtained from Hydrovas 10 (Bionap, Belpasso, Italy), to raw OP. Hydrovas 10, an OMWW commercial extract rich in hydroxytyrosol and tyrosol (total concentration 10%), was prepared according to the technique reported by Allouche et al. [14] and was dark stored at 4 °C before use.

The final concentration of hydroxytyrosol and tyrosol was adjusted to obtain 10 mg of phenols for 20 g of product, according to the “health claims” prescribed by the EU Rule 432/2012 for functional foods. 

### 2.2. Study Design and Participants

The participants were 98 healthy volunteers (M = 54, 55%), all students and active members of the CUS Salerno (Centro Universitario Sportivo), the Sports Center at the University of Salerno. A public notice was made to recruit healthy volunteers aged 18 to 25. Those who applied were interviewed to record age, gender, nutrition, and lifestyle habits, including the number of days of physical activity per week. Actual age mean and SD was 22.1 ± 1.8 years.

On this basis, two groups were formed: group A, including those who indicated a practice of three or more days of physical activity a week, and group B, including those who indicated a practice of just one or two days of physical activity a week. 

Each group was subsequently randomized into two subgroups, one for the dietary supplementation with ROP, and the other as the control group, with the OP.

All subjects gave their informed consent for inclusion before participation in the study. Anonymity was guaranteed as the data were depersonalized before being analyzed in aggregate form. The study was conducted in accordance with the Declaration of Helsinki [15], and the protocol was approved by the Ethics Committee of the University of Salerno (n. 21 of 2019-03-06).

### 2.3. Sample Collection and Biological Antioxidant Potential Measurement (BAP test)

After the randomization, a blood sample was drawn from the participants. An independent clinical laboratory trusted by the CUS Salerno was used. Participants were given the prescribed product (OP or ROP), sufficient for 30 days.

They were instructed to maintain their usual lifestyle and physical activity as reported at the interview and to add to their usual nutrition one tablespoon (20–25 grams) of product a day, without cooking it, for thirty days. For assistance purposes, contact details for a nutritionist trusted by the CUS Salerno were provided, but no request or call was made by any of the participants during the nutritional supplementation. At the end of the month, the participants returned for a second interview and sample.

To measure the antioxidant status and the effect of dietary supplementation, the biological antioxidant potential test (BAP) [16] was performed using a commercial kit (Diacron, Rome, Italy).

BAP results are expressed in micromoles of iron reduced per liter (μmol/L); the reference values in humans is considered as normal at for any level above the cutoff of 2200 μmol/L. As the linearity is reported excellent up to 10,000 μmol/L, the test can reliably capture any increment of antioxidant potential due to the dietary supplementation.

### 2.4. Statistical Analysis

The values at baseline for OP and ROP were their respective control groups, and OP was the control group for the ROP. The results were analyzed using R statistical software (Version 3.2.4), to perform descriptive statistics and compare data with the Student’s *t*-test, assuming that the difference was statistically significant for *p* < 0.05 and highly significant for *p* < 0.01.

## 3. Results

The BAP test showed an increase in antioxidant potential after supplementation (Table 1). There was a highly statistically significant difference with *p* < 0.01, for baseline vs. 30 days in all groups, notwithstanding the high SD, that can be attributed to the limited number of subjects and to the physiological variance of the antioxidants levels.

As expected, the increment obtained with ROP was higher than basic OP, and group B (low physical activity) showed a higher increment than group A ( high physical activity).

An unexpected finding was the one presented in Table 2. A comparison of smokers and non-smokers in the ROP group indicated there was an antioxidant increment clearly higher for smokers (*p* < 0.01). 

The smokers showed a mean BAP at baseline clearly lower than non-smokers, and the increment obtained during the 30 days of supplementation was nearly double for smokers than for non-smokers.

## 4. Discussion

The effects of dietary supplementation with an olive pâté reinforced with natural antioxidants recovered from olive mill waste have been studied. Our results showed that this dietary supplementation significantly increased the antioxidant levels and that this increment was significantly higher than that obtained using the basic olive pâté. Furthermore, we observed a significant effect of the supplementation in smokers, in which the antioxidant levels increased much more than in non-smokers.

### 4.1. Olive-Derived Antioxidants Health Effects

Our study confirms the hypothesis that nutrition enriched in natural antioxidants increases the levels of these beneficial molecules. There are randomized controlled trials that report beneficial effects in subjects with coronary artery disease [17] and in healthy overweight, in which the supplementation also helped weight loss [18]. The growing availability of data on the functional components of olive oil is improving the knowledge of its nutrigenomics [19] and nutritional products. This knowledge includes effects [20] on diseases and molecular characterization of individual compounds, especially phenols [21]. Fortification with phenols in many different foods and in particular with refined olive oil have been reported [22], but no experiments appear to have been performed until now using olive pâté. Thus, the present paper may be useful to propose the use of this product as a dietary supplement.

The use of olive pâté for dietary supplementation has been limited due to its limited availability. For centuries, it has been produced only in small quantities, usually handcrafted according to the ethnical traditions of the Mediterranean. In recent years, the development of the modern two-phase centrifuge [23] has generated large quantities of a by-product called “olive cake”, which is similar to the pâté because it is an olive paste devoid of woody parts. This by-product includes both lipophilic and hydrophilic fractions, has been tested for animal nutrition [24], and could potentially be used as a convenient source of phenolic compounds, with high content, long stability, and good effects. Until now, it has only been tested on cultured human fibroblasts [25], and no experiences in the human diet have been published so far.

### 4.2. Antioxidants Supplementation in Smokers and Physically Active People

The high increase in antioxidants in smokers is the most interesting finding of our work, which may deserve further studies. In fact, a study performed by Moschandreas et al. in 2002 [26] showed no difference between low- and high-phenol olive oil dietary supplementation. The main difference with the present study is in the phenol composition: Moschandreas et al. used an olive oil rich in oleuropein and ligstroside, with a low content of tyrosol and hydroxytyrosol. In contrast, the composition of the olive pâté used in this work is more rich and complex than oil and with supplementation of tyrosol and hydroxytyrosol. The latter could be the most relevant molecule in explaining the biological bases of our findings. In fact, an animal-model based study performed in 2000 [27] identified hydroxytyrosol supplementation as a protective factor in passive-smoke exposed subjects. It must be noted that in the studies mentioned above, the antioxidant outcome was measured using the tests available at that time, which were different from the ones available now. Unfortunately, no other studies of dietary supplementation of olive oil antioxidants on smokers appear to be available in literature, according to a 2013 systematic review of human studies on nutrition and oxidative stress [28], or to a literature search performed by us as of February 2018 using the query “dietary supplementation + olive oil + antioxidants + smokers”.

From another point of view, a study in 2000 [29] brings evidence that moderate supplementation with other antioxidants, i.e., ascorbic acid, in smokers with depleted antioxidants, restores their values to the same levels as non-smokers.

Our finding of a higher effect of supplementation in subjects with lower physical activity needs to be mentioned. It is known that regular exercise increases blood antioxidant capacity [11], so even in this case, the less active subjects could have received a higher benefit from dietary antioxidant supplementation. This finding may be paired with the fact that moderate exercise alone already has protective effects against oxidative stress in persons with impaired health [12,13].

### 4.3. Further Research Questions

Many research questions remain open, which could be considered as a research agenda for the future. The first is to confirm the finding of a difference in dietary supplementation sources in smokers. Is this just an effect of supplementation of phenols, or does the presence of the hydrophilic fraction in the pâté play a role? For instance, in a large study [30] on gastric cancer in 29,133 male smokers, some sources were associated with increased risk, while others seemed protective.

These studies on differences in the sources of antioxidants should be addressed by comparing oil and pâté supplementation, possibly not only in healthy volunteers.

Second is to investigate the difference between the effects of phenols in oil and in pâté, as well as the possible differences in specific categories of subjects for a number of biological actions of phenols, such as prevention of atherogenesis, cancer, anti-inflammation, and anti-microbial.

Third, the nutrigenomics and biological modeling of antioxidant action from olive pâté, given the lack of studies, appear as a research area nearly unexplored so far.

## 5. Conclusions

The use of olive pâté for nutritional purposes increases antioxidant levels, and that the reinforcement with phenols from OMWW improves this effect.

The reinforcement process itself may be very useful because it recovers phenols from OMWW from a “win–win” perspective: fewer phenols in the environment, for which they are a toxic waste, and more antioxidants in foodstuffs, for which they are a precious enrichment. Furthermore, a much higher diffusion of this improvement of the Mediterranean diet could be obtained if the pâté produced by the industrial process were available for human consumption, with possible lower costs and higher availability.

The effect of olive pâté may also be connected to different compositions because olive paste also contains parts of the pericarp and does not contain components from stone. Other factors to take into account may be connected to cultivar, maturation, and climate. 

Nutritional supplementation with reinforced olive pâté appears to be a useful way to increase the biological antioxidant potential and thus, promote health in the general population, and much more in smokers. Further studies would be very advisable.

## Figures and Tables

**Table 1:** Results of the biological antioxidant potential (BAP) test (μmol/L) for different clusters.

Cluster	BAP at Baseline		BAP at 30 Days		Mean Diff μmol/L	Mean Diff in % of Baseline
	Mean	SD	Mean	SD		
All the participants (n = 98)	3828.3	1368.0	4443.6	1524.9	615.3 *	16.0
Group A: (*n* = 49)	3824.7	1577.6	4356.7	1731.9	532.0 *	13.9
Group B: (*n* = 49)	3823.0	1137.2	4530.6	1297.8	698.6 *	18.2
ROP (*n* = 68)	3764.2	1446.8	4501.2	1591.2	736.9 *	19.5
OP (*n* = 30)	3973.7	1180.0	4313.2	1379.4	339.6	8.5

Group A = >=3 days activity/week; Group B = 1–2 days activity/week; ROP = reinforced olive patè; OP = olive patè without reinforcement; * = *p* < 0.01.

**Table 2:** Results of the BAP test (μmol/L) for smokers vs. non-smokers in the reinforced olive pâté (ROP) group.

	BAP at Baseline		BAP at 30 Days		Mean Diff μmol/L	Mean Diff % of Baseline
Participants Who Received Dietary Supplementation with ROP (*n* = 68)	Mean	SD	Mean	SD		
Smokers (*n* = 15)	3242.9	1606.6	4366.8	1884.1	1122.9 *	34.6
Non-smokers (*n* = 53)	3927.3	1387.7	4563.1	1521.3	635.7	16.2

* = *p* < 0.01.
